# The prevalence and risk factors for anxiety and depression symptoms among migrants in Morocco

**DOI:** 10.1038/s41598-023-30715-8

**Published:** 2023-03-06

**Authors:** Firdaous Essayagh, Meriem Essayagh, Sanah Essayagh, Ikram Marc, Germain Bukassa, Ihsane El otmani, Mady Fanta Kouyate, Touria Essayagh

**Affiliations:** 1 Faculté des Sciences Juridiques, Économiques et Sociales, Laboratoire Droit Privé et Enjeux de Développement, Université Sidi Mohamed Ben Abdellah, Fès, Morocco; 2 Office National de Sécurité Sanitaire des Produits Alimentaires, Oujda, Morocco; 3 Faculté des Sciences et Techniques, Laboratoire Agroalimentaire et Santé, Hassan First University of Settat, Settat, Morocco; 4 Institut Supérieur des Sciences de la Santé, Laboratoire Sciences et Technologies de la Santé, Hassan First University of Settat, Settat, Morocco; 5 Department of Indigenous Services Canada/Government of Canada, Health Surveillance and Assessment Unit, First Nations and Inuit Health, Regina, Saskatchewan Region Canada; 6 Unité Investigation et Recherche, Département de Surveillance à l’Agence Nationale de Sécurité Sanitaire, Conakry, Guinea

**Keywords:** Diseases, Risk factors

## Abstract

Humanitarian migration can result in mental health issues among migrants. The objective of our study is to determine the prevalence of anxiety and depression symptoms among migrants and their risk factors. A total of 445 humanitarian migrants in the Orientale region were interviewed. A structured questionnaire was used in face-to-face interviews to collect socio-demographic, migratory, behavioral, clinical, and paraclinical data. The Hospital Anxiety and Depression Scale was used to assess anxiety and depression symptoms. Risk factors for anxiety and depression symptoms were identified using multivariable logistic regression. The prevalence of anxiety symptoms was 39.1%, and the prevalence of depression symptoms was 40.0%. Diabetes, refugee status, overcrowding in the home, stress, age between 18 and 20 years, and low monthly income were associated with anxiety symptom. The associated risk factors for depression symptoms were a lack of social support and a low monthly income. Humanitarian migrants have a high prevalence of anxiety and depression symptoms. Public policies should address socio-ecological determinants by providing migrants with social support and adequate living conditions.

## Introduction

In recent decades, migration has increased to 281 million international migrants worldwide, accounting for 3.6% of the world's population^1^. Human migration has an impact on both developed and developing countries^2^. With 86,000 migrants in 2014^3^, Morocco, long known as a transit country to Europe, became a host country for migrants from West and Central Africa, as well as Syria^4^. Migration places people in situations that can have an impact on their physical, social, and mental health. The migration process can cause stress, feelings of loss, and social marginalization^5^, making migrants more vulnerable to health problems, including anxiety and depression. Depression is widely regarded as the leading cause of suicides and suicide attempts. Indeed, depression accounts for 40% to 80% of all suicide attempts globally^6^.

In 2017, anxiety disorders affected 260 million people worldwide, and depression affected 300 million, with economic consequences totaling at least $1000 billion (US) in lost productivity per year^7^. In 2009, a meta-analysis of populations exposed to conflict and refugees revealed a 30.8% prevalence of depression^8^. A systematic review of 8,176 resettled Syrian refugees in ten countries published in 2019 found a prevalence of anxiety of 26% and depression of 40%^9^.

To the best of our knowledge, no epidemiological study has focused on mental health among migrants in Morocco. Given the dynamics of migration and its impact on migrants' health, it is critical to investigate the prevalence of self-reported health issues among vulnerable populations. This would provide evidence on which policymakers in host countries and humanitarian organizations could rely to strengthen mental health services for undocumented migrants, asylum seekers, and refugees and meet their health needs in accordance with the Sustainable Development Goals and the global action plan "Promoting the health of refugees and migrants" (2019–2023)^9^.

Addressing mental health among undocumented migrants, asylum seekers, and refugees would also increase the chances of successful social integration, which would benefit the host country's socioeconomic capital in the long run. As a result, the objective of our study was to determine the prevalence of self-reported anxiety and depression symptoms among migrants in Morocco, as well as the risk factors associated with them.

## Methods

### Setting of the study, study design, and population

The Orientale Region covers an area of approximately 90,130 km^2^ and has a population of 2,314,346 people, or 6.8% of Morocco's total population^10^. The Mediterranean Sea borders it to the north, and Algeria borders it to the south. It is notable for its maritime coast, which stretches for 200 km. The region serves as a gateway to Africa, a focal point for the Maghreb, and a Mediterranean interface open to Europe. It is one of three major Moroccan regions that house asylum seekers and refugees.

Between November and December 2021, we conducted a cross-sectional survey among the migrant population that has relationships with associations working to improve the health of migrants in the Orientale region as stated in their statutes. The Prefecture of Oujda provided an exhaustive list of these associations. The sampling was divided into two stages. A random sample drawing was made to select the primary unit, consisting of 17 associations from the 30 associations present in Oujda. The secondary unit consisted of migrants aged 18 and older who were present in the Orientale region on the days of the survey and agreed to participate in the study. To determine the secondary unit in each selected primary unit, migrants were assigned a queuing number upon their arrival in the association, and these numbers were drawn at random to select the participants until the required sample size was achieved. A migrant was defined as any person of foreign origin in Morocco, regardless of their date of entry, duration of stay, or even settlement. The migrants were divided into three groups: (i) undocumented migrants are those who do not have a valid Moroccan residence permit; this situation could have resulted from entering the country without valid travel documents; being the child of undocumented parents; overstaying an entry visa; or losing a valid residence permit^11^; (ii) asylum seekers are people who are seeking safety from persecution or harm in a country other than their own and are waiting for a response to their application for refugee status; (iii) and refugees, who are defined as anyone who is recognized by the host country as being unable or unwilling to return to their country of origin due to a well-founded fear of persecution because of their race, religion, nationality, membership in a social group, or political beliefs^12^.

### Sample size determination

No previous study has measured the prevalence of self-reported anxiety and depression symptoms among migrants in Morocco. Hence, we used the hypothesis that the estimated prevalence of self-reported anxiety and depression symptoms is 50%. Considering the 95% confidence level, the 5% margin of error, and the 50% prevalence of self-reported anxiety and depression symptoms, the estimated minimum sample size was 384 migrants.

### Data collection

We collected data on the participant's socio-demographic background, behavioral habits, comorbidities, and paraclinical parameters using a standardized structured questionnaire during a face-to-face individual interview. The questionnaire was given in Arabic, French, or English, which are the main languages spoken by migrants in Morocco. The interviewers translated the questionnaire into French and English. The French-translated questionnaire was given to two community workers for retranslation into Arabic. These community agents were bilingual; French was their mother tongue, and Arabic their second language. This technique ensured that the French version and the version written in Arabic were identical. The same was true for the English language questionnaire, but this time with two community workers whose first language is English. To ensure data standardization, we trained and involved the same interviewers. The data were collected in a closed room to ensure confidentiality and anonymity. Participants were not compensated in order to reduce selection bias.

### Operational definitions

#### Anxiety symptom assessment

The WHO defines anxiety as a feeling of undetermined imminent danger accompanied by uneasiness, agitation, helplessness, and even annihilation^7^. In our study, participants self-rated their anxiety using the Hospital-Anxiety-and-Depression-Scale-A (HADS-A). On this scale, the participant reported how he or she felt over the previous two weeks^13^, this sub-scale consists of seven items: (i) I feel tense or irritable, (ii) I have a fear that something terrible will happen to me, (iii) I worry, (iv) I can sit quietly doing nothing and feel relaxed, (v) I have feelings of fear and my stomach is knotted, (vi) I am on the move and I can't keep it in place, and (vii) I have sudden feelings of panic. Each of these items was assigned a score ranging from 0 to 3, with "0" for no symptoms and "3" for the most severe symptoms. The sub-score for overall anxiety ranged from 0 to 21. Scores of 11 or higher were considered to signal elevated levels of anxiety symptomatology^13^.

#### Depression symptom assessment

Depression is defined as sadness, loss of interest or pleasure, feelings of guilt or low self-esteem, sleep or appetite disturbances, feeling tired and lack of concentration^7^. In our study, participants self-rated their depression using the Hospital-Anxiety-and-Depression-Scale-D subscale (HADS-D). The participant reported his or her feelings over the previous two weeks on this scale^13^.

This sub-scale consists of seven items: (i) I still enjoy the same things I used to, (ii) I laugh easily and see the bright side of things, (iii) I'm in a good mood, (iv) I feel like I'm idling, (v) I'm no longer concerned with how I look, (vi) I look forward to doing certain things, and (vii) I enjoy a good book as well as a good radio or television show. Each of these items was assigned a score ranging from 0 to 3, with "0" for no symptoms and "3" for the most severe symptoms. The sub-score for overall depression ranged from 0 to 21. Scores of 11 or higher were considered to signal elevated levels of depression symptomatology.

Our study's variables included sociodemographic, migratory, behavioral, and clinical characteristics.

Social support was reported when a participant stated receiving assistance from close friends or associations.

The consumption of alcohol and tobacco was reported when the participant declared having consumed them during the last two months preceding the survey.

Overweight and obesity have been categorized according to the WHO body mass index classification^14,15^.

The stress was reported when the participant declared being stressed.

The countries of origin of migrants have been divided into two groups: Sub-Saharan Africa and the Eastern Mediterranean Region.

### Data management and statistical analysis

Epi Info version 7.2.0.1 was used to enter and analyze data. The precautionary measures to protect the confidentiality of the collected information and the anonymity of the participants were strictly followed. All tests were two-sided, with statistical significance set at less than 0.05.

Continuous variables were expressed as mean and standard deviation, while categorical variables were expressed as numbers and percentages. In bivariable analysis, the proportions of categorical variables were compared using the Pearson chi-2 test or, where applicable, Fisher's exact test. Where applicable, continuous variables were compared using the analysis of variance test or the Mann–Whitney test. We included in the multiple logistic regression any variables with a *p*-value up to 0.05 in the bivariable analysis.

We used a multiple logistic regression procedure to determine the full model. We revealed the association between each risk factor and the presence of symptoms of anxiety or depression using the adjusted odds ratio (AOR) and its 95% confidence interval (CIs).

### Ethics approval and consent to participate

The study adhered to the Helsinki Declaration. Potential participants were informed of the study's main goal and procedure. All subjects who took part in the study signed an informed written statement of consent. The study protocol was reviewed and approved by the ethical review board of the faculty of Medicine and Pharmacy in Rabat, Morocco (#33/21).

## Results

### Socio-economic, demographic, and migration-specific characteristics

Table 1 summarizes the socio-economic, demographic, and migration-specific characteristics. During the study period, 445 participants were recruited, with 174 (39.1%) reporting anxiety symptoms and 178 (40.0%) reporting depression symptoms. The participants’ average age was 27.9 ± 10.9 years, ranging from 18 to 73 years, with 306 (68.8%) males. There were 288 (51.2%) undocumented migrants, 177 asylum seekers (39.8%) and 40 refugees (9.0%). A total of 109 (24.5%) participants were homeless; 40 (11.9%) lived in households with more than ten people; 300 (67.4%) had a low monthly income; and 221 (49.7%) had no social support.

**Table 1:** Socio-demographic and migrant characteristics, Morocco, 2021.

	All participants n (%)	Anxiety symptoms n (%)	Absence of anxiety symptoms n (%)
Total participants	445 (100)	174 (39.1)	271 (60.9)
Mean age in years ± sd	27.9 ± 10.9	28.1 ± 12.3	27.8 ± 09.9
Age group in years			
18–20	119 (26.7)	58 (33.3)	61 (22.5)
21–25	120 (27.0)	39 (22.4)	81 (29.9)
26–30	85 (19.1)	33 (19.0)	52 (19.2)
31 and older	121 (27.2)	44 (25.3)	77 (28.4)
Sex			
Male	306 (68.8)	126 (72.4)	180 (66.4)
Female	139 (31.2)	48 (27.6)	91 (33.6)
Marital status			
Partnered^†^	140 (31.5)	57 (32.8)	83 (30.6)
Single^‡^	305 (68.5)	117 (67.2)	188 (69.4)
Education			
Illiterate	112 (25.2)	47 (27.0)	65 (24.0)
Elementary	144 (32.4)	57 (32.8)	87 (32.1)
Middle school	56 (12.6)	22 (12.6)	34 (12.5)
High school	92 (20.6)	37 (21.3)	55 (20.3)
College	41 (09.2)	11 (06.3)	30 (11.1)
Native country			
Eastern Mediterranean Region	100 (22.5)	46 (26.4)	54 (20.0)
Sub-Saharan Africa	345 (77.5)	128 (73.6)	217 (80.0)
Length of stay in Morocco (in years)			
≥ 5	94 (21.1)	39 (22.4)	55 (20.3)
< 5	351 (78.9)	135 (77.6)	216 (79.7)
Legal status			
Refugee	40 (09.0)	30 (17.2)	10 (03.7)
Asylum seeker	177 (39.8)	73 (42.0)	104 (38.4)
Undocumented migrant	228 (51.2)	71 (40.8)	157 (57.9)
Number of countries crossed (n = 430)			
≥ 3	134 (31.2)	56 (33.3)	78 (29.8)
< 3	296 (68.8)	112 (66.7)	184 (70.2)
Housing type			
Homeless	109 (24.5)	47 (27.0)	62 (22.9)
House*	336 (75.5)	127 (73.0)	209 (77.1)
Number of persons per house (n = 336)			
≥ 10	40 (11.9)	25 (19.8)	15 (07.1)
[5–9]	174 (51.8)	63 (50.0)	111 (52.9)
≤ 4	122 (36.3)	38 (30.2)	84 (40.0)
Occupation			
No	430 (96.6)	170 (97.7)	260 (96.0)
Yes	15 (03.4)	4 (02.3)	11 (04.0)
Monthly income ($)			
≤ 150	300 (67.4)	140 (80.5)	160 (59.0)
> 150	145 (32.6)	34 (19.5)	111 (41.0)
Health insurance			
No	444 (99.8)	173 (99.4)	271 (100.0)
Yes	1 (00.2)	1 (00.6)	0 (00.0)
Social support			
No	221 (49.7)	79 (45.4)	142 (52.4)
Yes	224 (50.3)	95 (54.6)	129 (47.6)

	All participants n (%)	Depression symptoms n (%)	Absence of depression symptoms n (%)
Total participants	445 (100)	178 (40.0)	267 (60.0)
Mean age in years ± sd	27.9 ± 10.9	26.7 ± 11.8	28.7 ± 10.3
Age group in years			
18–20	119 (26.7)	61 (34.3)	58 (21.7)
21–25	120 (27.0)	52 (29.2)	68 (25.5)
26–30	85 (19.1)	28 (15.7)	57 (21.3)
31 and older	121 (27.2)	37 (20.8)	84 (31.5)
Sex			
Male	306 (68.8)	132 (74.2)	174 (65.2)
Female	139 (31.2)	46 (25.8)	93 (34.8)
Marital status			
Partnered^†^	140 (31.5)	56 (31.5)	84 (31.5)
Single^‡^	305 (68.5)	122 (68.5)	183 (68.5)
Education			
Illiterate	112 (25.2)	36 (20.2)	76 (28.5)
Elementary	144 (32.4)	74 (41.6)	70 (26.2)
Middle school	56 (12.6)	28 (15.7)	28 (10.5)
High school	92 (20.6)	27 (15.2)	65 (24.3)
College	41 (09.2)	13 (07.3)	28 (10.5)
Native country			
Eastern Mediterranean region	100 (22.5)	43 (24.2)	57 (21.3)
Sub-Saharan Africa	345 (77.5)	135 (75.8)	210 (78.7)
Length of stay in Morocco (in years)			
≥ 5	94 (21.1)	30 (16.8)	64 (24.0)
< 5	351 (78.9)	148 (83.2)	203 (76.0)
Legal status			
Refugee	40 (09.0)	8 (04.5)	32 (12.0)
Asylum seeker	177 (39.8)	43 (24.2)	134 (50.2)
Undocumented migrant	228 (51.2)	127 (71.3)	101 (37.8)
Number of countries crossed (n = 430)			
≥ 3	134 (31.2)	56 (32.4)	78 (30.4)
< 3	296 (68.8)	117 (67.6)	179 (69.6)
Housing type			
Homeless	109 (24.5)	66 (37.1)	43 (16.1)
House*	336 (75.5)	112 (62.9)	224 (83.9)
Number of persons per house (n = 336)			
≥ 10	40 (11.9)	19 (17.1)	21 (09.3)
[5–9]	174 (51.8)	60 (54.1)	114 (50.7)
≤ 4	122 (36.3)	32 (28.8)	90 (40.0)
Occupation			
No	430 (96.6)	176 (98.9)	254 (95.1)
Yes	15 (03.4)	2 (01.1)	13 (04.9)
Monthly income ($)			
≤ 150	300 (67.4)	148 (83.1)	152 (56.9)
> 150	145 (32.6)	30 (16.9)	115 (43.1)
Health insurance			
No	444 (99.8)	177 (99.4)	267 (100.0)
Yes	1 (00.2)	1 (00.6)	0 (00.0)
Social support			
No	221 (49.7)	131 (73.6)	90 (33.7)
Yes	224 (50.3)	47 (26.4)	177 (66.3)

*sd* standard deviation. *House means living in house or apartment or reception center. ^†^A partnered means to be married or to be in concubine. ^‡^Single means to be single or divorced or widower.

### Behavioral characteristics

The data in Table 2 show that 96 (21.6%) of the participants used tobacco, 72 (16.2%) drank alcohol, 44 (9.9%) were physically inactive, 320 (71.9%) were stressed, and 18 (4.0%) were diabetic.

**Table 2:** Behavioral characteristics and clinical features of migrants, Morocco, 2021.

	All participants n (%)	Anxiety symptoms (n = 174)	Absence of anxiety symptoms (n = 271)
Tobacco consumption			
Yes	96 (21.6)	43 (24.7)	53 (19.6)
No	349 (78.4)	131 (75.3)	218 (80.4)
Alcohol consumption			
Yes	72 (16.2)	29 (16.7)	43 (15.9)
No	373 (83.8)	145 (83.3)	228 (84.1)
Physical activity			
Unsatisfactory	44 (09.9)	28 (16.1)	16 (05.9)
Satisfactory	401 (90.1)	146 (83.9)	255 (94.1)
Stress			
Yes	320 (71.9)	145 (83.3)	175 (64.6)
No	125 (28.1)	29 (16.7)	96 (35.4)
Comorbidity			
Yes	15 (03.4)	10 (05.8)	5 (01.8)
No	430 (96.6)	164 (94.2)	266 (98.2)
Overweight/obesity			
Yes	177 (39.8)	68 (39.1)	109 (40.2)
No	268 (60.2)	106 (60.9)	162 (59.8)
Diabetes			
Yes	18 (04.0)	13 (07.5)	5 (01.9)
No	427 (96.0)	161 (92.5)	266 (98.1)
Hypertension			
Yes	124 (27.9)	41 (23.6)	83 (30.6)
No	321 (72.1)	133 (76.4)	188 (69.4)

	All participants n (%)	Depression symptoms (n = 178)	Absence of depression symptoms (n = 267)
Tobacco consumption			
Yes	96 (21.6)	20 (11.2)	76 (28.5)
No	349 (78.4)	158 (88.8)	191 (71.5)
Alcohol consumption			
Yes	72 (16.2)	10 (05.6)	62 (23.2)
No	373 (83.8)	168 (94.4)	205 (76.8)
Physical activity			
Unsatisfactory	44 (09.9)	16 (09.0)	28 (10.5)
Satisfactory	401 (90.1)	162 (91.0)	239 (89.5)
Stress			
Yes	320 (71.9)	94 (52.8)	226 (84.6)
No	125 (28.1)	84 (47.2)	41 (15.4)
Comorbidity			
Yes	15 (03.4)	04 (02.2)	11 (04.1)
No	430 (96.6)	174 (97.8)	256 (95.9)
Overweight/obesity			
Yes	177 (39.8)	63 (35.4)	114 (42.7)
No	268 (60.2)	115 (64.6)	153 (57.3)
Diabetes			
Yes	18 (04.0)	5 (02.8)	13 (04.9)
No	427 (96.0)	173 (97.2)	254 (95.1)
Hypertension			
Yes	124 (27.9)	51 (28.6)	73 (27.3)
No	321 (72.1)	127 (71.4)	194 (72.7)

### Anxiety symptoms

In Table 1, a total of 174 (39.5%) of the 445 participants screened showed symptoms of anxiety. Their average age was 28.1 ± 12.3 years, 126 (72.4%) were male and 48 (27.6%) were female. According to the socio-demographic data, 73 (42.0%) were asylum seekers, 71 (40.8%) were undocumented migrants, and 30 (17.2%) were refugees. 170 participants (97.7%) were unemployed, and 140 (80.4%) had a low monthly income.

The behavioral characteristics reported in Table 2 show that: 28 (16.1%) of participants reported inadequate physical activity; 145 (83.3%) reported stress; and 10 (5.7%) reported comorbidities. Diabetes was reported in 13 (7.5%) of the participants.

The cut-off *p*-value after the bivariable analysis was set at a *p*-value ≤ 0.05. The legal status of undocumented migrants was considered the reference group during the bivariable analyses. This choice is based on the assumption that undocumented migrants are a population that still hopes for a better future in the host country after their legal status has been regularized. On the other hand, refugees are a population who witnesses the persistence of difficulties in accessing basic services even after regularization of their administrative situation, this realization leads to a higher level of anxiety or depression than that of undocumented migrants and asylum seekers. As mentioned in Table 3, according to the bivariable analysis, we identified eight factors associated with self-reported anxiety symptoms: (i) age between 18 and 20 years compared to 31 years or older (OR of 1.66; 95% CI [0.99–2.78] (ii) refugee compared to undocumented migrant (OR of 6.63; 95% CI [3.07–14.3]); (iii) living with more than ten people per household compared to four people or fewer each household (OR of 3.68; 95% CI [1.74–7.76]); (iv) low monthly income (OR of 2.85; 95% CI [1.82–4.46] (v) unsatisfactory physical activity (OR of 3.05; 95% CI [1.60–5.83]); (vi) stress (OR of 2.74; 95% CI [1.71–4.38]); (vii) presence of comorbidity (OR of 3.24; 95% CI [1.08–9.65]); and (viii) having diabetes (OR of 4.29; 95% CI [1.50–12.2]).

**Table 3:** Multivariable analysis (odds ratio, *p-value*) of risk factors associated with anxiety symptoms among migrants, Morocco, 2021.

	Bivariable analysis		Multivariable analysis complete model	
	COR (95% CI)	*p-*value	AOR (95% CI)	*p-*value
Age group in years				
18–20/ ≥ 31	1.66 [0.99–2.78]	0.05	3.56 [1.55–8.16]	0.002
21–25/ ≥ 31	0.84 [0.49–1.43]	0.52	1.14 [0.54–2.39]	0.79
26–30/ ≥ 31	1.11 [0.62–1.96]	0.71	1.76 [0.84–3.66]	0.12
Legal status				
Refugee/undocumented migrant	6.63 [3.07–14.3]	< 0.001	6.26 [2.34–16.7]	0.0003
Asylum seeker/undocumented migrant	1.55 [1.03–2.33]	0.03	1.38 [0.75–2.55]	0.29
Number of persons per house				
≥ 10/ ≤ 4	3.68 [1.74–7.76]	0.0006	4.08 [1.70–9.76]	0.001
5–9/ ≤ 4	1.25 [0.76–2.05]	0.36	1.28 [0.72–2.27]	0.38
Monthly income ≤ 150 ($)	2.85 [1.82–4.46]	< 0.001	3.64 [2.08–6.37]	< 0.001
Unsatisfactory physical activity	3.05 [1.60–5.83]	0.0005	2.02 [0.90–4.53]	0.08
Stress	2.74 [1.71–4.38]	< 0.001	4.30 [1.88–9.83]	0.0005
Having comorbiditie	3.24 [1.08–9.65]	0.03	0.75 [0.18–3.07]	0.69
Having diabetes	4.29 [1.50–12.2]	0.003	18.6 [2.80–124.4]	0.002

*COR* Crude odds ratio, *AOR* Adjusted odds ratio, *CI* Confidence interval.

### Depression symptoms

In Table 1, a total of 178 (40.0%) of the 445 participants screened showed symptoms of depression. Their average age was 26.7 ± 11.8 years, 132 (74.2%) were males and 46 (25.8%) were females. According to socioeconomic data, 127 (71.3%) were undocumented migrants, 43 (24.2%) were asylum seekers, and 8(4.5%) were refugees. 139 (78.1%) participants were from Sub-Saharan Africa, and, 148 (83.1%) had a low monthly income. According to Table 2, 131 (73.6%) participants reported no social support, while 94 (52.8%) reported stress.

The cut-off *p*-value after the bivariable analysis was set at *p*-value ≤ 0.05. As mentioned in Table 4, according to the bivariable analysis, we identified 16 factors associated with the self-reported depression symptoms: (i) age between 18 and 20 years compared to 31 years or older (OR of 2.38; 95% CI [1.40–4.04]); (ii) age between 21 and 25 years compared to 31 years or older (OR of 1.73; 95% CI [1.02–2.94]); (iii) male sex (OR of 1.53; 95% CI [1.00–2.23]); (iv) elementary level of education compared to college (OR of 2.27; 95% CI [1.09–4.74]); (v) middle school level of education compared to college (OR of 2.15; 95% CI [0.92–4.99]); (vi) refugee compared to undocumented migrant (OR of 0.19; 95% CI [0.08–0.45]); (vii) asylum seeker (OR of 0.25; 95% CI [0.16–0.39]); (viii) homeless (OR of 3.06; 95% CI [1.96–4.79]); (ix) number of people per household more than or equal to ten compared to four people or fewer each household (OR of 2.54; 95% CI [1.21–5.33]); (x) number of people per household between five and nine compared to four people or fewer each household (OR of 1.48; 95% CI [0.88–2.46]); (xi) unemployment (OR of 4.50; 95% CI [1.00–20.1]); (xii) low monthly income (OR of 3.73; 95% CI [2.35–5.91]); (xiii) lack of social support (OR of 5.48; 95% CI [3.60–8.33]); (xiv) tobacco consumption (OR of 0.31; 95% CI [0.18–0.54]); (xv) alcohol consumption (OR of 0.19; 95% CI [0.09–0.39]); and (xvi) stress (OR of 0.20; 95% CI [0.13–0.31]).

**Table 4:** Multivariable analysis (odds ratio, *p-value*) of risk factors associated with depression symptoms among migrants, Morocco, 2021.

	Bivariable analysis		Multivariable analysis complete model	
	COR (95% CI)	*p-*value	AOR (95% CI)	*p-*value
Age group in years				
18–20/ ≥ 31	2.38 [1.40–4.04]	0.001	1.01 [0.42–2.41]	0.96
21–25/ ≥ 31	1.73 [1.02–2.94]	0.04	1.64 [0.79–3.40]	0.17
26–30/ ≥ 31	1.11 [0.61–2.02]	0.71	1.57 [0.73–3.36]	0.23
Sex male/female	1.53 [1.00–2.33]	0.04	0.85 [0.46–1.56]	0.60
Education				
Illiterate/college	1.02 [0.47–2.19]	0.95	1.89 [0.60–5.94]	0.27
Elementary/college	2.27 [1.09–4.74]	0.02	1.41 [0.46–4.33]	0.54
Middle school/college	2.15 [0.92–4.99]	0.07	1.29 [0.35–4.73]	0.69
High school/college	0.89 [0.40–1.98]	0.78	2.13 [0.65–6.91]	0.20
Legal status				
Refugee/undocumented migrant	0.19 [0.08–0.45]	0.0001	0.39[0.13–1.21]	0.10
Asylum seeker/undocumented migrant	0.25 [0.16–0.39]	< 0.001	0.63 [0.32–1.21]	0.16
Homeless	3.06 [1.96–4.79]	< 0.001	0.0 [0.00- > 1.0^E12^]	0.96
Number of persons per house				
≥ 10/ ≤ 4	2.54 [1.21–5.33]	0.01	1.57 [0.63–3.93]	0.32
5–9/ ≤ 4	1.48 [0.88–2.46]	0.13	1.47 [0.79–2.73]	0.21
Unemployed	4.50 [1.00–20.1]	0.04	2.08 [0.39–11.0]	0.38
Monthly income ≤ 150 ($)	3.73 [2.35–5.91]	< 0.001	2.72 [1.50–4.91]	0.0009
Lack of social support	5.48 [3.60–8.33]	< 0.001	3.48 [1.82–6.64]	0.0001
Tobacco consumption	0.31 [0.18–0.54]	< 0.001	0.71 [0.32–1.56]	0.40
Alcohol consumption	0.19 [0.09–0.39]	< 0.001	0.33 [0.13–0.82]	0.01
Stress	0.20 [0.13–0.31]	< 0.001	0.66 [0.30–1.41]	0.28

*COR* Crude odds ratio, *AOR* Adjusted odds ratio, *CI* Confidence interval.

### Multivariable analysis

After adjusting for the other variables, we identified the following factors as risk factors associated with self-reported anxiety symptoms: (i) having diabetes (AOR of 18.68; 95% CI [2.80–124.42]) ; (ii) refugee compared to undocumented migrants (AOR of 6.26; 95% CI [2.34–16.8]); (iii) living with more than ten people per household compared to four people or fewer each household (AOR of 4.08; 95% CI [1.70–9.76]); (iv) stress (AOR of 4.30; 95% CI [1.88–9.83]); (v) age between 18 and 20 years compared to 31 years or older (AOR of 3.56; 95% CI [1.55–8.16]); and (vi) low monthly income (AOR of 3.64; 95% CI [2.08–6.37])“Table 3”.

For the depression symptoms, after adjustment for the other variables, two risk factors were associated: (i) lack of social support (AOR of 3.48; 95% CI [1.82–6.64]); and (ii) low monthly income (AOR of 2.72; 95% CI [1.50–4.91]). Alcohol consumption was a protective factor for depression symptoms (AOR of 0.33; 95% CI [0.13–0.82]) “Table 4”.

## Discussion

To the best of our knowledge, this is Morocco's first study on the self-reported prevalence of anxiety and depression symptoms among migrants. In the literature, community assessment tools for mental diseases exist, but the emphasis tends to be on trauma and resilience factors or as a component part of "quality of life" rating scales. The Hospital Anxiety and Depression Score, which was not originally intended for community use, has been widely used in community settings around the world, including in our study^16,17^. The prevalence of self-reported anxiety symptoms among undocumented migrants, refugees, and asylum seekers in our survey was 39.1%, which is similar to Richter's study in 2018, where the prevalence of anxiety was 39.2% among 296 migrants^18^. However, it remains higher than the 4.7% reported in Nepal out of 574 migrants^19^ and the 7.7% reported in Lebanon out of 194 migrants^12^.

For self-reported symptoms of depression, the prevalence was 40.0% in our survey, which is consistent with the 39.5% reported in Turkey on 238 migrants^20^. However, it is still lower than the 53.4% reported in the Kizilhan study on 296 migrants in 2018^21^.

In our study, the young age of migrants was associated with self-reported anxiety symptoms. This could be explained by discrimination, feelings of social isolation, a lack of financial resources, and uncertainty about the future. The discussion carried out with migrants had shown that the young subjects who left their country, their comfort zone, for a new country, came with enormous expectations and a lot of energy and motivation, but once in the host country, they came up against a reality marked by the difficulties of rapid access to adequate employment^22^. This situation pushed migrants to want to seek answers to the reasons for their unemployment, and it is then that migrant began to devalue themselves and loose self-esteem, which would lead to anxiety^23^.

Refugee status was associated with self-reported anxiety symptoms in our study. Indeed, we know that migrants, whether refugees, asylum seekers, or undocumented migrants, are a vulnerable population whose mental health can suffer. They may have been victims of traumatic events such as war, the loss of a family member, or physical and/or sexual violence before their arrival in the host country^4,22^, all of which can lead to anxiety and depressive disorders. Added to this is the negative perception of migrants that the population of the host country has, the cumbersomeness of the administrative procedures, and the length of the regularization procedures^24^ Once granted a refugee status, migrants find themselves caught between an ideal discourse on asylum and the reality in the host country. They come up against the persistence of discrimination, difficulties in accessing health services, access to work, adequate housing, and professional deskilling. All of these are factors that lead to the onset of anxiety symptoms in refugees.

In our study, low monthly income was associated with self-reported symptoms of anxiety and depression among migrants. Indeed, the low monthly income limits access to basic necessities such as housing, food, and medical care.

According to the literature, the presence of social support is associated with a low level of mental disorders. It facilitates migrants' integration into the host country and overcomes isolation and anti-migrant sentiment^25^. A lack of social support was associated with self-reported depression symptoms in our study.

Stress, considered a risk factor for several diseases, particularly anxiety and depression, could be linked to migrants' awareness that they have lost everything, that they no longer have any control over certain aspects of their lives, and that they have no social status in the host country^26^. It could also be the result of structural barriers caused by migrants' ignorance of the services available to them in the host countries. In addition, there is the fear of stigmatization, cultural and linguistic differences, post-migration living conditions, and the migrant's legal status in the host country. Dissatisfaction with the healthcare system can also be a source of stress and a major challenge for migrants. A healthcare system that does not provide services tailored to cultural differences can be a source of stress and an impediment to the well-being of migrants. In our study, stress was associated with self-reported anxiety symptoms.

We know that physical activity, when practiced in sufficient quantity, can improve an individual's health. It has the potential to prevent the onset of mental disorders such as anxiety and depression. According to the literature, people who engage in regular physical activity have a high level of life satisfaction^27^. Wieland's 2015 study of 127 migrants found that a lack of physical activity was associated with the development of mental disorders^28^. The disruption of daily life, economic pressure, ignorance of the environment, loss of social network, and lack of motivation in the face of extreme stress and uncertainty about the future could be the main barriers to the practice of physical activity among migrants. In our study, unsatisfactory activity physique was not associated with self-reported anxiety or depression.

Overcrowding and insufficient housing have long been associated with mental health issues, including among humanitarian migrants as a result of financial constraints, discrimination, and housing policies. In our study, living in overcrowded environments was associated with self-reported anxiety symptoms. A similar result was reported from a study of 681 migrants in Sweden^29^. This may be explained by the lack of privacy, autonomy, and isolation from the local community.

In terms of conditions, diabetes was associated with self-reported anxiety symptoms in our study. Indeed, these chronic illnesses necessitate medical attention and medication. Failure to meet these needs due to migrants' limited resources can exacerbate their health problems^22^, including mental health.

In our study, alcohol consumption was a protective factor against self-reported depression among migrants. The literature review showed that light to moderate alcohol consumption can provide short-term relief from self-reported depressive symptoms due to its euphoric and stimulating effects^30^. It gives migrants a feeling of relief, distancing themselves from their problems, and allows them to feel a tranquilizing or soothing effect. Excessive alcohol consumption allows them to anesthetize the emotions that assail them and that they are unable to manage and express. They drink to forget their ill-being, to endure their suffering, or even to get to sleep. However, in the long term, chronic consumption can become a triggering factor or aggravator of the depressive state^30^.

## Limitations of the study

Despite the guarantee of anonymity and the use of community actors during data collection, our study had some limitations, including prevarication bias in relation to monthly income and behavior characteristics during data collection.

Although the sampling suggests the presence of a selection bias in the study, which includes only migrants attending associations, this is not entirely correct. Indeed, in the Orientale region, the associations are well-known for the assistance and support they provide in terms of access to food, health care, and other basic life necessities for vulnerable people. As a result, they are frequented by refugees, asylum seekers, undocumented migrants, and even newly arrived migrants.

## Conclusion

Undocumented migrants, refugees, and asylum seekers are vulnerable populations to mental illnesses. The findings of our study revealed a high prevalence of self-reported anxiety and depression symptoms. Self-reported anxiety symptoms were associated with factors such as diabetes, young age, refugee status, crowding, low monthly income, and stress. A lack of social support and a low income were associated with self-reported depression symptoms. Following these findings, it is critical to emphasize the importance of preventing mental disorders among migrants by addressing social, economic, and environmental determinants. Future research measuring mental health status at arrival then at six months/12-month intervals would be beneficial to track changes.

## Data Availability

All data generated or analyzed during this study are included in this published article.
